# Medium and long-term aortic remodeling following aortic valve replacement combined with anticoagulation for DeBakey I aortic dissection

**DOI:** 10.3389/fcvm.2025.1575757

**Published:** 2025-05-09

**Authors:** Yuanyuan Li, Shifeng Yang, Congshan Ji, Haoyuan Yang, Jinshu Sun, Juncheng Jiang, Ximing Wang, Long Wang

**Affiliations:** ^1^ Department of Radiology, Shandong Provincial Hospital Affiliated to Shandong First Medical University, Jinan, China; ^2^ Department of Cardiovascular Surgery, Shandong Provincial Hospital Affiliated to Shandong First Medical University, Jinan, China; ^3^ Department of Cardiovascular Surgery, Qilu Hospital of Shandong University, Jinan, China

**Keywords:** aortic dissection, DeBakey type I, anticoagulation, vascular remodeling, thrombosis

## Abstract

**Background:**

Total arch replacement with frozen elephant trunk has achieved promising outcomes for DeBakey type I aortic dissection. However, the effects of anticoagulation on the distal false lumen and unfavorable remodeling of the distal aorta after aortic valve replacement remains insufficiently understood. This study aimed to assess the impact of anticoagulation following aortic valve replacement on medium and long-term vascular remodeling outcomes in DeBakey type I aortic dissection.

**Methods:**

We conducted a retrospective analysis of patients who underwent total arch replacement with a frozen elephant trunk for DeBakey Type I aortic dissection from September 2013 to December 2024. Seventy-two patients with preoperative and at least six months postoperative aortic computed tomography angiography images were included and stratified into a valve replacement group (*n* = 30) and a non-valve replacement group (*n* = 42). Various parameters of the residual dissected aorta were analyzed at six specific levels to evaluate late aortic remodeling, aortic diameter, and false lumen thrombosis.

**Results:**

The median follow-up period was 17 (interquartile range IQR = 9–27) months. Preoperative characteristics and complications did not significantly differ between the two groups, except for body mass index, blood pressure, and severity of aortic regurgitation. The valve replacement group had longer cardiopulmonary bypass time, aortic cross-clamping time, cardiac arrest time, larger trunk diameter, and higher intraoperative red blood cells transfusion volume compared to the non-valve replacement group. However, there were no statistically significant differences in concomitant procedures, postoperative complications, or length of hospital stay. Regarding postoperative changes in the diameter of aortic lumen and true lumen, there were statistically significant difference in the true lumen on level 1 and the aortic lumen on level 3–5 of the valve replacement group. Additionally, the aortic lumen and true lumen on level 1 and true lumen on level 2 of the non-valve replacement group were statistically difference. There were no significant differences in the rate of aortic remodeling at each level or overall between the two groups. The postoperative false lumen thrombosis rate was higher in the mid-descending thoracic aorta and lower in the distal abdominal aorta.

**Conclusions:**

Anticoagulation following aortic valve replacement for Debakey I aortic dissection has been shown to influence aortic diameter and the false lumen thrombosis rate, but it does not significantly affect the aortic remodeling rate. Overall, anticoagulation appears to be a viable treatment strategy for Debakey I aortic dissection.

## 1 Introduction

Type A aortic dissection (TAAD), particularly Debakey type I aortic dissection, stands out as one of the most critical cardiovascular emergencies due to its extensive nature, carrying an hourly mortality rate of 1%–2% in untreated patients (1, 2). Therefore, emergency surgical intervention is often recommended in clinical practice. In recent years, Sun's procedure has emerged as first-line therapy for TAAD, demonstrating significant short-term efficacy, with early mortality rates decreasing from 11%–36% to 6%–8% (3, 4). Depending on the extent of aortic root involvement, mechanical valve-based aortic valve replacement is employed clinically when the dissection affects the aortic sinus and aortic valve, leading to moderate to severe valvular insufficiency (4). Advancements in surgical techniques have contributed to improved patient outcomes, yet the long-term management of residual dissections remains challenging.

It is generally understood that lifelong anticoagulation with warfarin sodium is required following aortic valve replacement, which theoretically affects postoperative distal thrombosis in false lumen (FL) and indirectly leads to pathologic aortic remodeling (5, 6). Residual aortic dissection poses the risk of progressive aortic dilation, rupture, or secondary aortic surgery (2, 7), so systematic follow-up with computed tomography angiography (CTA) is essential to mitigate long-term adverse postoperative events, including aortic lumen (AL) expansion, true lumen (TL) compression, critical limb ischemia, and aortic rupture (5, 8). While early and mid-term clinical outcomes and aortic remodeling in TAAD have been documented previously (9–11), there remains a paucity of data regarding medium and long-term postoperative aortic remodeling. Furthermore, comparative studies focusing specifically on aortic valve replacement vs. non-valve replacement techniques for Debakey type I aortic dissection are scarce. Herein, our objective was to determine the efficacy anticoagulation after aortic valve replacement on medium and long-term vascular remodeling outcomes in DeBakey type I aortic dissection.

## 2 Materials and methods

### 2.1 Study population

**Figure 1:**
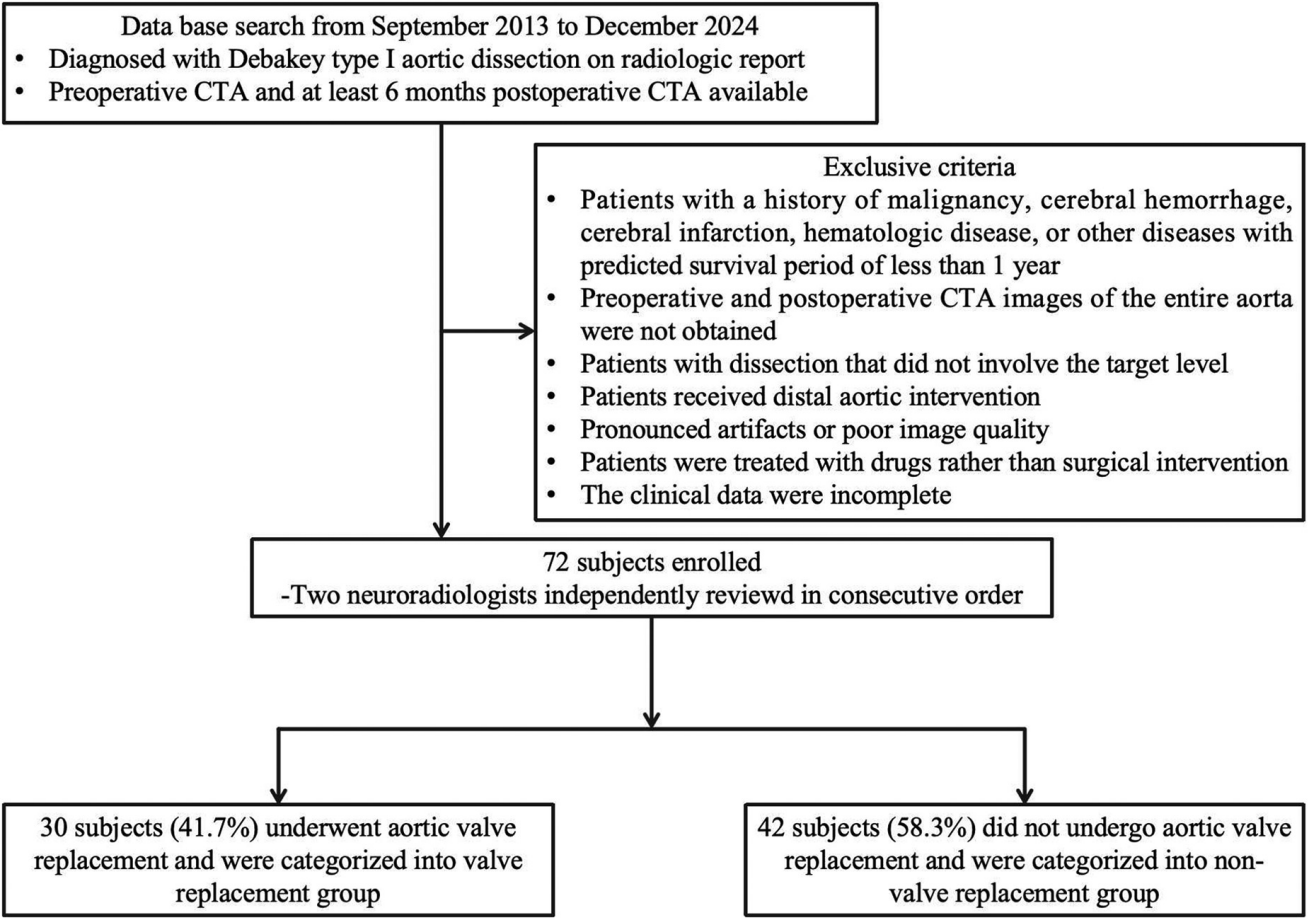
Flowcharts of the patient selection criteria.

The protocol for this single-center, retrospective, observational study was approved by the ethical committee of Shandong Provincial Hospital (Shandong, China) (approval number: 20201-361), and the individual written informed consent was waived. Patients diagnosed with Debakey type I aortic dissection who underwent Sun's procedure at our center from September 2013 to December 2024 were retrospectively reviewed. All patients underwent preoperative CTA and had at least 6 months of follow-up data available. The flowchart of the patient-selection criteria is detailed in Figure 1. Eventually, this study enrolled a total of 72 subjects (male: 50; female: 22; mean age: 48.5 years ± 9.9), who were stratified into valve replacement group (*n* = 30) and non-valve replacement group (*n* = 42). Patients' comprehensive clinical records were also fully extracted from the clinical database, encompassing demographics, clinical characteristics, clinical outcomes, and surgical details.

### 2.2 Acquisition technique and reconstruction

Retrospective ECG-gated CTA examinations were conducted with dual-source CT scanner (Definition Flash, Siemens Healthcare, Forchheim, Germany), and the scanning parameters and contrast agent injection protocols were used according to the previous literature (12). Prior to surgery, all patients underwent thoracoabdominal aortic CTA and echocardiography to ascertain the type of dissection, the location of the entry tear, aortic diameters, involvement of the aortic valve and branches, which served as foundational information for the surgical approach. The location of the primary entry tear was identified on the preoperative CTA, and was verified during the operation as well. Subsequently, postoperative CTA scan was conducted at least 6 months after surgery to evaluate mid to long-term progression, including changes in aortic diameters, the thrombosis status of FL in descending aortic diameter between the two groups. Images were generally reconstructed through specialized image processing program (syngo.via, SIEMENS Healthineers). This image analysis software automatically delineates the boundaries of enhanced vessels, employing a semi-automatic centerline algorithm to measure key aortic parameters along the vertical axis using the centerline as a reference. Aortic measurements were performed by two independent observers after reviewing 10 cases (about 14% of the cohort), and interobserver correlations were recorded.

### 2.3 Cross-sectional image analysis of remodeling

Based on morphological measurements from the preoperative and postoperative CTA images, we evaluated mid- to long-term aortic remodeling in residual descending aortic dissections. Morphological features of aortic remodeling included changes in aortic diameter at selected measurement levels (expansion of the TL and shrinkage of the FL) and formation of FL thrombosis. Cumulative occurrence rates of aortic remodeling at each level were indirectly calculated through changes in aortic diameter.

**Figure 2:**
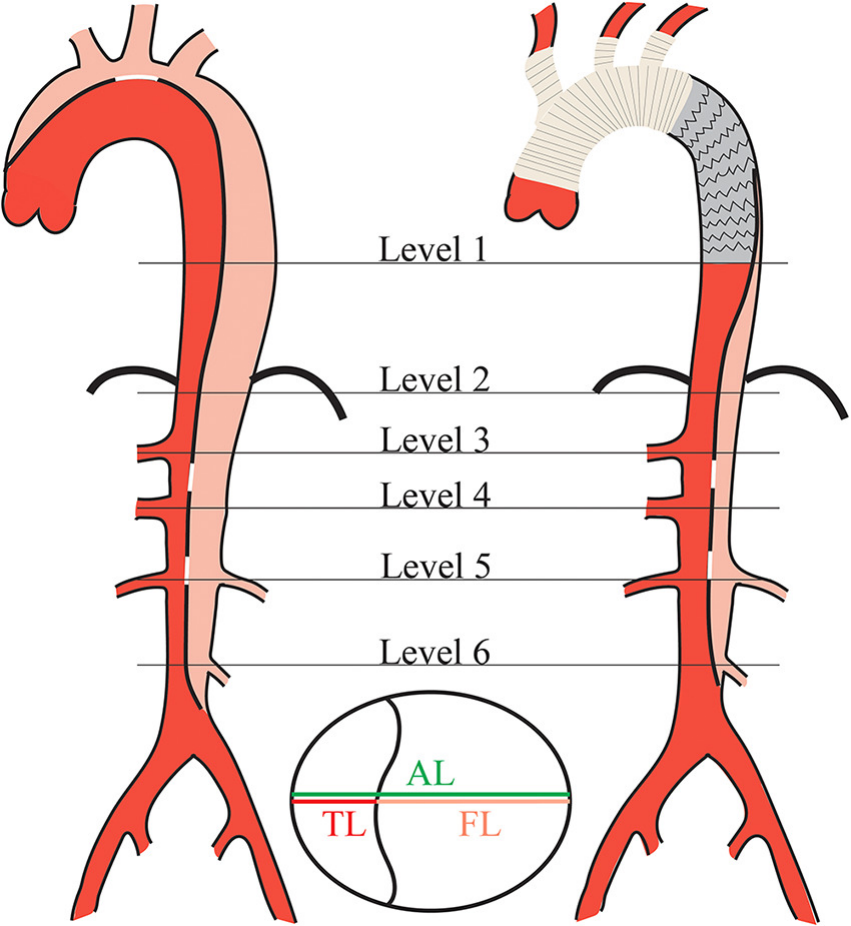
Schematic diagram of aortic diameter measurement in DeBakey type I aortic dissection pre- and post-surgery. Aortic diameters measurement was taken at standardized location with respect to the aortic anatomy in each level. AL, aortic lumen; TL, true lumen; FL, false lumen.

TL and FL diameters were measured on axial CT images perpendicular to the contour of the intimal flap, and the measurement criteria was the distance between the inner edges of the transverse section perpendicular to the aortic axis (13). To clearly describe the distal state of the residual dissection, aortic diameters were measured at the following specific anatomical levels (14): (1) Level 1 (L1): distal end of the stent graft; (2) Level 2 (L2): just at the level of diaphragm; (3) Level 3 (L3): proximal abdominal aorta at the level of the celiac trunk artery; (4) Level 4 (L4): abdominal aorta at the level of the superior mesenteric artery; (5) Level 5 (L5): abdominal aorta at the level of the renal artery; (6) Level 6 (L6): distal aorta at the level of the inferior mesenteric artery. Preoperative and postoperative aortic diameter measurement schematic diagram is shown in Figure 2.

The progression of aortic dissection was evaluated using the Society for Vascular Surgery/Society of Thoracic Surgeons (SVS/STS) Aortic Dissection Classification System (15). According to the SVS/STS system, the extent of dissection involvement is defined as the length of the intimal tear along the centerline of the aorta. The diameter of the primary entry was determined as the maximum distance between the flap margins of the inner intimal sheet on the axial image obtained through multi-planar reconstructions (12). Aortic tortuosity was quantified as the ratio of the centerline length to the linear distance from the aortic arch (at the level of the left subclavian artery) to the end of the abdominal aorta (16). TL compression was defined as the TL having a diameter of less than 5 mm at least at three different levels.

In this study, aortic remodeling was categorized as positive, negative, or stable. The criteria for defining aortic remodeling were consistent with those used in previous studies (3, 5, 17): positive remodeling was defined if there was significant decrease in AL diameter and/or increase in TL diameter, stable if there were no significant changes in AL or TL diameters, and negative if there was significant increase in AL diameter or decrease in TL diameter. Notably, changes in AL or TL diameter greater than 10% were considered significant.

The patency status of the FL is an important predictor of regional aortic growth and reintervention rates. Therefore, assessing the extent of FL thrombosis is necessary. Partial or complete thrombosis may be overestimated based on arterial phase images alone. All patients underwent three-phase CT scans during follow-up, and the degree of thrombosis in different levels of downstream FL was comprehensively evaluated using arterial or venous phase images. The degree of FL thrombosis includes no thrombosis, partial thrombosis, malabsorption and complete obliteration (17, 18). No thrombosis was defined as complete opacification of the entire length of the FL. Partial thrombosis was characterized by obvious contrast agent presence within the FL with some degree of thrombus formation. Malabsorption and complete obliteration indicated absence of contrast agent within the FL on arterial or delayed images.

### 2.4 Surgical procedures

All patients with aortic dissection received routine medical treatment since admission, including absolute bed rest, blood pressure control, heart rate reduction, sedation, analgesia, etc. According to the results of preoperative thoracoabdominal aortic CTA, echocardiography, and intraoperative aortic root exploration of the aortic root: For patients without involvement of the aortic valve by the dissection tear, aortic root repair and Sun's procedure were performed; For patients with preoperative aortic valve disease complicated by dissection or dissection-induced moderate to severe aortic valve insufficiency, Sun's procedure combined with aortic valve replacement is performed. All surgeries were performed by two experienced surgeons. The specifics of Sun's procedure have been detailed in previous studies (19), involving replacement of the aortic arch and elephant trunk technique, using a covered stent graft to cover the primary tear in the dissection, isolating the FL's main blood flow and restoring blood supply to the TL. The stent length and diameter were selected based on preoperative CTA, and the stent is implanted and sutured to the distal end of the four-branched artificial graft during the operation. The time from symptom onset to surgery, cardiopulmonary bypass time, aortic cross-clamping time, cardiac arrest time, type of stent graft, landing zone position of the stent's distal end, concomitant procedures, intraoperative blood transfusions, postoperative complications, and length of hospital stay were recorded.

Following valve replacement, patients typically take oral warfarin sodium once daily, with a dosage ranging from 2.5 to 5 mg. The dose of warfarin varies significantly between individuals. During anticoagulation therapy, regular monitoring of the International Normalized Ratio (INR) is required to dynamically adjust the warfarin dosage, with an INR target range of 1.5–2.0. Generally, INR levels should be monitored more frequently during the early stages of treatment to ensure effective anticoagulation while avoiding excessive bleeding. The INR was considered to be stable when the INR remains within the target range with minimal fluctuation for 2–3 consecutive measurements without dosage adjustments.

### 2.5 Statistical analysis

All statistical analyses were performed using GraphPad Prism 10 (GraphPad Software Inc., CA, USA) and SPSS 27.0 (SPSS Inc., Chicago, IL, USA) software packages. The normality of data distribution was verified using the one-sample Kolmogorov–Smirnov test. Continuous variables were compared between groups using independent sample *t*-test, one-way analysis of variance or non-parametric Mann–Whitney *U* test. Categorical variables were compared between groups using the chi-square test *P* < 0.05 was considered statistically significant between the data. The Kappa test was used to assess agreement between two physicians regarding the classification of branch vessel involvement, thrombosis status, and rupture location. A Kappa value >0.81 was considered excellent agreement. The Bland-Altman method was used to assess the consistency in measuring diameter and length between the two physicians.

## 3 Results

### 3.1 Patient characteristics and baseline information

During the follow-up period, a total of 72 patients diagnosed with DeBakey I aortic dissection were included and analyzed. The median follow-up duration was 17 months in both groups, with IQR: 10–26 months in valve replacement group and IQR: 8–27 months in non-valve replacement group. Additionally, 66% of patients undergoing imaging examinations at least 1 year after surgery. Table 1 displays demographic characteristics, comorbidities, and laboratory data of the patients. The study cohort predominantly consisted of males (69.4%) with a mean age of 48.5 ± 9.9 years. The non-valve replacement group had a higher body mass index (BMI) compared to the valve replacement group, with a statistically significant difference (*p* = 0.020). There were no statistically significant differences in terms of age, gender, heart rate, smoking history, and drinking history between the two groups. There were no significant differences between the study cohorts in terms of clinical symptoms.

**Table 1:** Baseline demographics and clinical variables.

Variables	Whole cohort (*n* = 72)	Valve replacement group (*n* = 30)	Non-valve replacement group (*n* = 42)	*p* value
Age (year), mean ± SD	48.5 ± 9.9	46.7 ± 10.4	49.7 ± 9.4	0.551
Male gender, *n* (%)	50 (69.4%)	20 (66.7%)	30 (71.4%)	0.796
BMI (kg/m^2^), mean ± SD	25.1 ± 3.2	24.0 ± 3.2	25.8 ± 3.1	0.020*
Heart rate, mean ± SD	82.1 ± 11.2	81.0 ± 11.3	82.9 ± 11.2	0.484
Smoking, *n* (%)	21 (29.2%)	10 (33.3%)	11 (26.2%)	0.602
Drinking, *n* (%)	23 (31.9%)	11 (36.7%)	12 (28.6%)	0.609
Comorbidity				
Hypertension, *n* (%)	49 (68.1%)	15 (50.0%)	34 (81.0%)	0.010*
Hypercholesterolaemia, *n* (%)	11 (15.3%)	4 (13.3%)	7 (16.7%)	0.753
Diabetes, *n* (%)	4 (5.6%)	1 (3.3%)	3 (7.1%)	0.636
CAD, *n* (%)	5 (6.9%)	1 (3.3%)	4 (9.5%)	0.393
CVD, *n* (%)	2 (2.8%)	0	2 (4.8%)	0.507
CKD, *n* (%)	1 (1.4%)	1 (3.3%)	0	0.417
Marfan syndrome, *n* (%)	3 (4.2%)	3 (10.0%)	0	0.068
Bicuspid aortic valve, *n* (%)	5 (6.9%)	4 (13.3%)	1 (2.4%)	0.153
Symptoms				
Chest pain, *n* (%)	63 (87.5%)	28 (93.3%)	35 (83.3%)	0.289
Back pain, *n* (%)	31 (43.1%)	12 (40.0%)	19 (45.2%)	0.810
Abdominal pain, *n* (%)	21 (29.2%)	10 (33.3%)	11 (26.2%)	0.602
Headache or coma, *n* (%)	3 (4.2%)	2 (6.7%)	1 (2.4%)	0.567
Limb pain, *n* (%)	6 (8.3%)	4 (13.3%)	2 (4.8%)	0.227

Note: Data are presented as the mean ± standard deviation (SD) or *n* (%). BMI, body mass index; SBP, systolic blood pressure; DBP, diastolic blood pressure; CAD, coronary artery disease; CVD, cerebrovascular disease; CKD, chronic kidney disease.

*Denotes a significant difference between the samples (*p* < 0.05).

### 3.2 Preoperative and postoperative imaging features

Detailed CTA and echocardiography characteristics of baseline aortic dissection anatomy are shown in Table 2. There were no statistically significant differences between the two groups in terms of the location and diameter of the primary entry tear, number of tears, length of aortic dissection, aortic tortuosity. Additionally, within the entire cohort, both groups exhibited signs of complex dissections such as branches originating from the FL, annular tear, compression of the TL, and malperfusion, but these signs did not differ significantly between the two groups. Regarding the extent of involvement in aortic dissection, the most common locations affected in the distal dissection were the common iliac artery (27.8%) and the external iliac artery (33.3%).

**Table 2:** Preoperative and postoperative imaging features.

Variables	Whole cohort (n = 72)	Valve replacement group (*n* = 30)	Non-valve replacement group (*n* = 42)	*p* value
Location of primary entry tear				
Aortic root, *n* (%)	19 (26.0%)	9 (30.0%)	10 (23.8%)	0.596
Ascending aorta, *n* (%)	22 (30.6%)	11 (36.7%)	11 (26.2%)	0.438
Transverse arch, *n* (%)	25 (34.7%)	7 (23.3%)	18 (29.0%)	0.132
Proximal descending aorta, *n* (%)	6 (8.3%)	3 (10.0%)	3 (7.1%)	0.688
Diameter of primary entry tear (mm), mean ± SD	16.2 ± 10.1	18.5 ± 10.6	14.6 ± 9.6	0.108
Number of tears, mean ± SD	4.8 ± 1.5	4.9 ± 1.6	4.7 ± 1.5	0.592
Dissection extends to abdominal branches				
Coeliac trunk, *n* (%)	4 (5.6%)	2 (6.7%)	2 (4.8%)	>0.999
SMA, *n* (%)	5 (6.9%)	3 (10.0%)	2 (4.8%)	0.643
RA, *n* (%)	10 (13.9%)	3 (10.0%)	7 (16.7%)	0.506
Distal abdominal aorta, *n* (%)	9 (12.5%)	2 (6.7%)	7 (16.7%)	0.289
Common iliac artery, *n* (%)	20 (27.8%)	12 (40.0%)	8 (19.0%)	0.065
External iliac artery, *n* (%)	24 (33.3%)	8 (26.7%)	16 (38.1%)	0.447
Branches originating form FL, mean ± SD	3.1 ± 1.8	3.3 ± 1.9	3.0 ± 1.6	0.411
Malperfusion, *n* (%)	12 (16.7%)	5 (16.7%)	7 (16.7%)	>0.999
Myocardial ischemia, *n* (%)	2 (2.8%)	1 (3.3%)	1 (2.4%)	>0.999
Cerebral ischemia, *n* (%)	5 (6.9%)	1 (3.3%)	4 (9.5%)	0.393
Visceral ischemia, *n* (%)	3 (4.2%)	2 (6.7%)	1 (2.4%)	0.567
Renal failure, *n* (%)	5 (6.9%)	3 (10.0%)	2 (4.8%)	0.643
Lower extremity ischemia, *n* (%)	5 (6.9%)	1 (3.3%)	4 (9.5%)	0.393
Aortic regurgitation				
Mild, *n* (%)	22 (30.6%)	5 (16.7%)	17 (40.5%)	0.039*
Moderate, *n* (%)	26 (36.1%)	12 (40.0%)	14 (33.3%)	0.623
Severe, *n* (%)	15 (20.8%)	13 (43.3%)	2 (4.8%)	0.000*
LVEF (%), mean ± SD	60.3 ± 2.4	59.6 ± 3.0	60.7 ± 1.7	0.051
Length of aortic dissection(mm), mean ± SD	558.8 ± 96.6	561.6 ± 95.1	556.9 ± 98.8	0.840
Tortuosity, mean ± SD	1.2 ± 0.1	1.1 ± 0.1	1.2 ± 0.1	0.164
Annular tear, *n* (%)	28 (38.9%)	13 (43.3%)	15 (35.7%)	0.625
Preoperative TL compression, *n* (%)	8 (11.1%)	5 (16.7%)	3 (7.1%)	0.265
Postoperative TL compression, *n* (%)	2 (2.8%)	1 (3.3%)	1 (2.4%)	>0.999
Preoperative maximum descending aorta diameter (mm), mean ± SD	30.6 ± 4.7	30.1 ± 4.0	30.9 ± 5.1	0.505
Postoperative maximum descending aorta diameter (mm), mean ± SD	30.6 ± 4.8	31.2 ± 4.7	30.1 ± 4.9	0.364

Note: Data are presented as the mean ± standard deviation (SD) or *n* (%). SMA, superior mesenteric artery; RA, renal artery; TL, true lumen; FL, false lumen; LVEF, left ventricular ejection fraction.

*Denotes a significant difference between the samples (*p* < 0.05).

Preoperative routine echocardiography was performed to assess aortic valve regurgitation and left ventricular ejection fraction (LVEF). Both groups had normal LVEFs (mean 60.3% ± 2.4%), with no statistically significant difference. Aortic regurgitation occurred in both groups. However, there were significantly more severe aortic regurgitation (*p* = 0.000) and fewer mild aortic regurgitation (*p* = 0.039) in the valve-replacement group than in the non-valve replacement group. Preoperative assessment of aortic valve regurgitation also provide basis for the establishment of surgical strategy in this study.

The observers demonstrated good consistency in the involvement of branch vessels (*k* = 0.886), degree of thrombosis (*k* = 0.898), and primary entry tear location (*k* = 0.866). The Bland-Altman test results showed good consistency between the measurements of the two observers. The deviations for the AL diameter, TL diameter, involvement length, and primary entry tear diameter were 0.100, 0.150, 0.155, and 0.075, respectively, with 95% confidence intervals (CI) of 2.472 to −2.272, 1.020 to −0.720, 1.066 to −0.756, and 0.618 to −0.468.

### 3.3 Operative details

Surgical details for the entire cohort are presented in Table 3. The average time from onset of symptoms to surgery was 8.8 ± 14.1 days in the valve replacement group and 7.6 ± 13.7 days in the non-valve replacement group, with no statistically significant difference between the groups. All surgeries were completed successfully. In the valve replacement group, the cardiopulmonary bypass time, aortic cross-clamping time, cardiac arrest time, size of trunk, and intraoperative red blood cell transfusion were 239.8 ± 55.0 min, 145.0 ± 41.5 min, 26.8 ± 14.4 min, 26.2 ± 1.2 mm, and 6.4 ± 3.4 units, respectively. These above values were significantly greater than those in the non-valve replacement group, with statistical significance. All patients had successful stent implant, with the most common distal anchoring position of the stent being the upper thoracic segment (T6–T7) in 56.6% of the valve replacement group and 61.9% of the non-valve replacement group, with no statistically significant difference between the groups.

**Table 3:** Operative variables.

Variables	Valve replacement group (*n* = 30)	Non-valve replacement group (*n* = 42)	*p* value
Onset to surgery (days), mean ± SD	8.8 ± 14.1	7.6 ± 13.7	0.711
CPB time (min), mean ± SD	239.8 ± 55.0	196.5 ± 35.7	0.0001*
Aortic cross-clamping time (min), mean ± SD	145.0 ± 41.5	97.5 ± 18.2	<0.0001*
Cardiac arrest time (min), mean ± SD	26.8 ± 14.4	21.0 ± 8.0	0.035*
Size of trunk (mm), mean ± SD	26.2 ± 1.2	25.3 ± 1.0	0.001*
Site of distal anastomosis			
Upper thoracic segment (T6–T7), *n* (%)	17 (56.6%)	26 (61.9%)	0.808
Mid-thoracic segment (T8–T10), *n* (%)	13 (43.3%)	16 (38.1%)	0.808
Concomitant procedure, *n* (%)	8 (26.7%)	6 (14.3%)	0.234
CABG, *n* (%)	5 (16.7%)	3 (7.1%)	0.265
Mitral valvuloplasty, *n* (%)	1 (3.3%)	0	0.417
Tricuspid valvuloplasty, *n* (%)	1 (3.3%)	0	0.417
Carotid subclavian bypass, *n* (%)	0	2 (4.8%)	0.510
Renal artery PTA, *n* (%)	1 (3.3%)	1 (2.4%)	>0.999
Caesarean section, *n* (%)	1 (3.3%)	0	0.417
Blood transfusion			
Red blood cells (unit), mean ± SD	6.4 ± 3.4	4.0 ± 2.4	0.001*
Fresh frozen plasma (ml), mean ± SD	623.0 ± 225.4	599.3 ± 300.5	0.716
Platelets (unit), mean ± SD	1.5 ± 0.6	1.4 ± 0.6	0.377
Postoperative complications, *n* (%)	2 (6.7%)	7 (16.7%)	0.289
Stroke, *n* (%)	0	3 (7.1%)	0.261
Lower extremity ischemia, *n* (%)	0	3 (7.1%)	0.261
Anastomotic leak, *n* (%)	2 (6.7%)	1 (2.4%)	0.567
Hospital days (days), mean ± SD	21.6 ± 10.9	19.8 ± 9.2	0.453

Note: Data are presented as the mean ± standard deviation (SD) or *n* (%). CPB, cardiopulmonary bypass; CABG, coronary artery bypass grafting; PTA, percutaneous transluminal angioplasty; NA, not applicable.

*Denotes a significant difference between the samples (*p* < 0.05).

Additionally, concomitant procedures were performed in 14 patients (19.4%): in the valve replacement group, 1 patient underwent simultaneous coronary artery bypass grafting (CABG) and tricuspid valvuloplasty, 4 patients underwent CABG, 1 patient underwent mitral valvuloplasty, 1 patient underwent renal artery percutaneous transluminal angioplasty (PTA), and 1 patient underwent caesarean section; in the non-valve replacement group, 3 patients underwent CABG, 1 patient underwent ascending aortic-femoral bypass, 1 patient underwent carotid subclavian bypass, and 1 patient underwent renal artery PTA. There were no statistically significant differences between the groups in terms of concomitant procedures, postoperative complications, or length of hospital stay.

### 3.4 Aortic remodeling parameters

Changes in aortic diameters for patients with aortic dissection are presented in Table 4. Comparing the preoperative and postoperative diameters of the AL and TL in the two groups, there was an overall increasing trend in both the AL and TL diameter except at the distal level of the stent (i.e., Level L1). Additionally, the degree of change in the proximal level of the AL and TL diameter was greater than that in the distal level. In relation to the increase in TL diameter, the increase in ascending aorta AL diameter was less favorable for remodeling. Regarding postoperative changes in the diameter of AL and TL, there were statistically significant difference in the TL diameter on level 1 and the AL diameter on level 3–5 of the valve replacement group. Additionally, the AL and TL diameter on level 1 and TL diameter on level 2 of the non-valve replacement group were statistically difference.

**Table 4:** Changes in diameter of the AL and TL.

Level of aorta	Group	AL PO (mm)	AL FU (mm)	*p* value	TL PO (mm)	TL FU (mm)	*p* value
Level 1	Group A	29.6 ± 2.8	27.9 ± 5.6	0.133	10.8 ± 4.1	21.9 ± 6.2	<0.0001*
	Group B	30.1 ± 4.6	26.4 ± 5.1	0.0003*	11.2 ± 5.0	23.3 ± 4.6	<0.0001*
Level 2	Group A	27.0 ± 4.5	29.1 ± 4.6	0.066	10.2 ± 4.7	11.2 ± 5.1	0.456
	Group B	27.6 ± 4.7	28.7 ± 4.9	0.274	11.4 ± 4.1	15.0 ± 6.0	0.002*
Level 3	Group A	24.9 ± 1.9	27.6 ± 3.9	0.001*	9.1 ± 4.4	9.9 ± 4.6	0.528
	Group B	26.3 ± 3.9	27.4 ± 4.1	0.217	10.8 ± 4.1	12.3 ± 5.1	0.122
Level 4	Group A	23.6 ± 2.0	25.5 ± 4.3	0.036*	10.1 ± 5.3	10.6 ± 4.5	0.694
	Group B	24.6 ± 3.2	25.8 ± 4.6	0.187	11.3 ± 4.5	11.9 ± 5.0	0.554
Level 5	Group A	20.8 ± 2.3	22.6 ± 4.1	0.048*	9.3 ± 5.3	9.8 ± 4.8	0.706
	Group B	21.7 ± 2.8	22.9 ± 4.0	0.119	10.8 ± 4.4	10.6 ± 4.6	0.871
Level 6	Group A	20.5 ± 3.8	21.7 ± 4.4	0.267	10.6 ± 7.4	9.5 ± 5.1	0.512
	Group B	19.6 ± 2.6	20.4 ± 3.9	0.269	11.8 ± 5.4	11.3 ± 5.3	0.645

Note: Data are presented as the mean ± standard deviation (SD). Group A, valve replacement group; Group B, non-valve replacement group; AL, aortic lumen; TL, true lumen; PO, preoperative; FU, follow up.

*Denotes a significant difference between the samples (*p* < 0.05).

**Figure 3:**
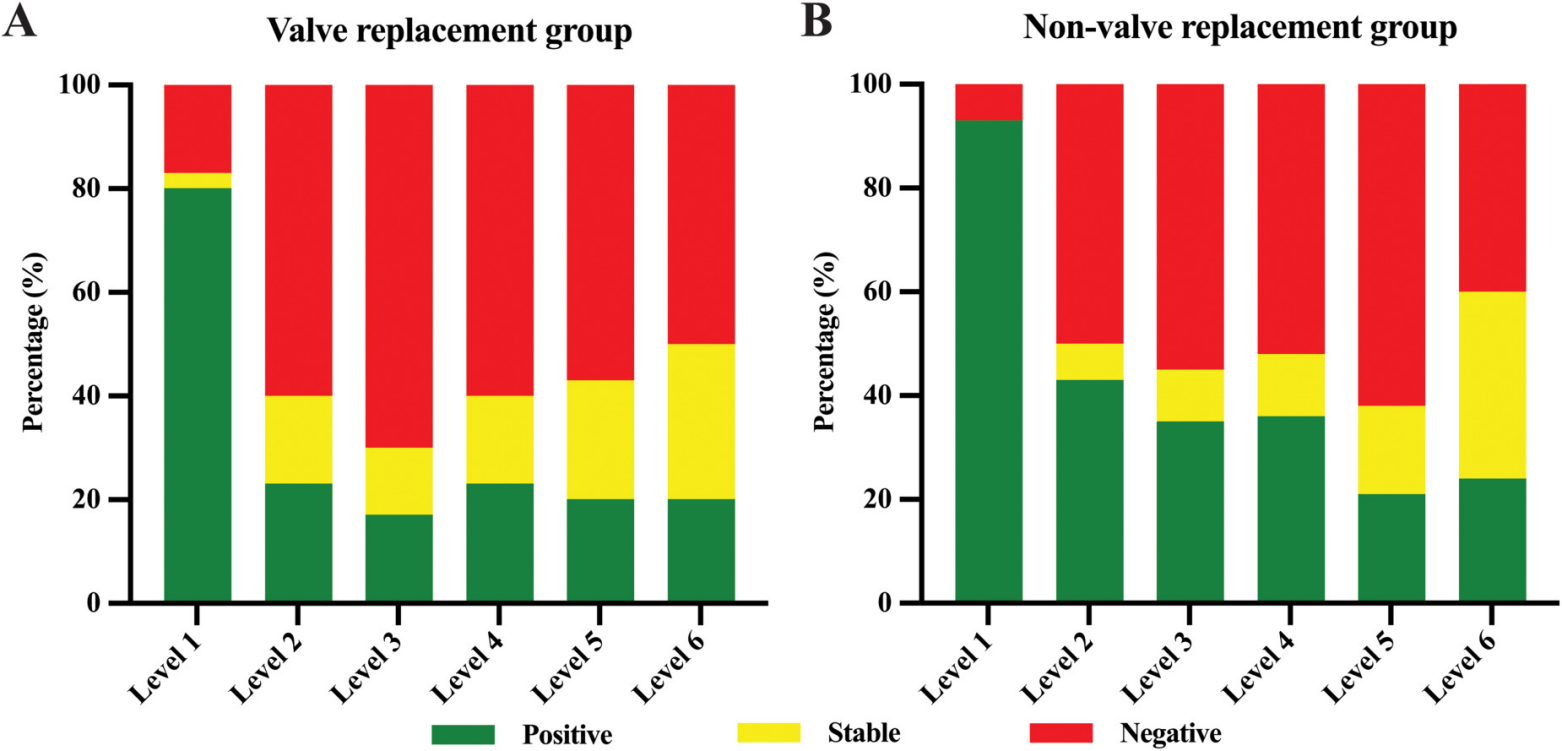
Aortic remodeling rate of valve replacement group **(A)** and non-valve replacement group **(B)**. Green represents positive remodeling, yellow represents stable, and red represents negative remodeling.

According to the proposed classification of aortic remodeling, aortic remodeling rate calculations were performed on 72 patients with aortic dissection. Aortic remodeling rates and comparisons between the two groups are shown in Table 5 and Figure 3. Positive remodeling was more common at the aortic level adjacent to the stent (i.e., Level L1), with rates of 80.0% in the valve replacement group and 92.9% in the non-valve replacement group. As distance from the stent increased, the degree of positive remodeling gradually decreased, with negative remodeling becoming more common than positive remodeling. Comparison of aortic remodeling rates between the two groups of showed that although the valve replacement group showed fewer instances of positive remodeling and more instances of negative remodeling at levels distant from the stent compared to the non-valve replacement group, there was no statistically significant difference in aortic remodeling rates between the groups at each level.

**Table 5:** Aortic remodeling rates for the entire cohort.

Level of aorta	Positive			Stable			Negative		
	Group A (%)	Group B (%)	*p* value	Group A (%)	Group B (%)	*p* value	Group A (%)	Group B (%)	*p* value
Level 1	24 (80.0%)	39 (92.9%)	0.151	1 (3.3%)	0	0.417	5 (16.7%)	3 (7.1%)	0.265
Level 2	7 (23.3%)	18 (42.9%)	0.132	5 (16.7%)	3 (7.1%)	0.265	18 (60.0%)	21 (50.0%)	0.475
Level 3	5 (16.7%)	15 (35.7%)	0.110	4 (13.3%)	4 (9.5%)	0.711	21 (70.0%)	23 (54.8%)	0.227
Level 4	7 (23.3%)	15 (35.7%)	0.307	5 (16.7%)	5 (11.9%)	0.732	18 (60.0%)	22 (52.4%)	0.632
Level 5	6 (20.0%)	9 (21.4%)	>0.999	7 (23.3%)	7 (16.7%)	0.553	17 (56.7%)	26 (61.9%)	0.808
Level 6	6 (20.0%)	10 (23.8%)	0.780	9 (30.0%)	15 (35.7%)	0.800	15 (50.0%)	17 (40.5%)	0.476
Total	12	19	0.810	—			18	23	0.810

Note: Data are presented as *n* (%). Group A (*n* = 20), valve replacement group; Group B (*n* = 42), non-valve replacement group.

**Figure 4:**
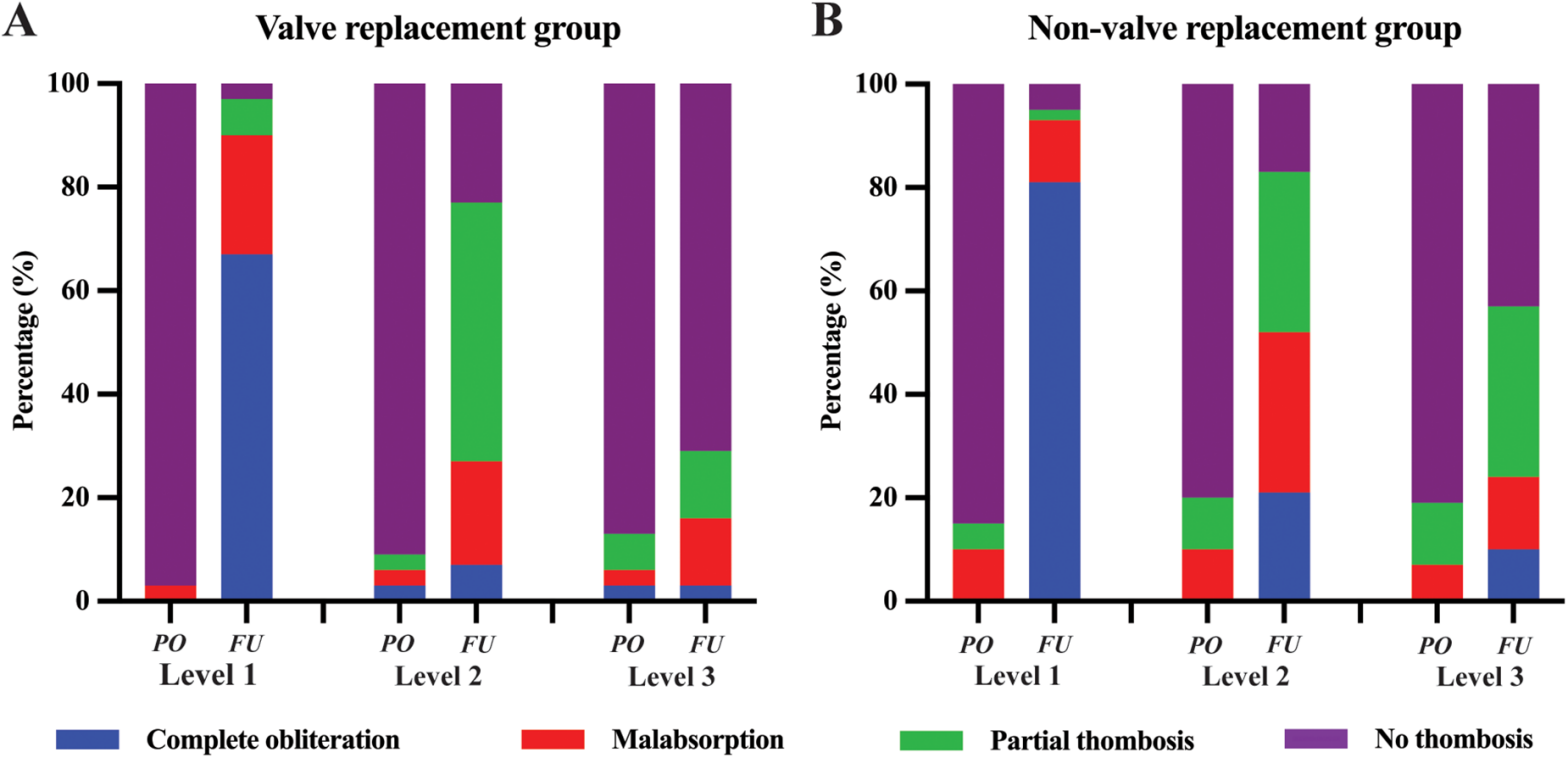
The degree of FL thrombosis in valve replacement group **(A)** and non-valve replacement group **(B)**. The complete obliteration (blue), malabsorption (red), partial thrombosis (green), or no thrombosis (purple) status of FL at the Level 1, Level 2, and Level 3. FL, false lumen; PO, preoperative. FU, follow up.

**Figure 5:**
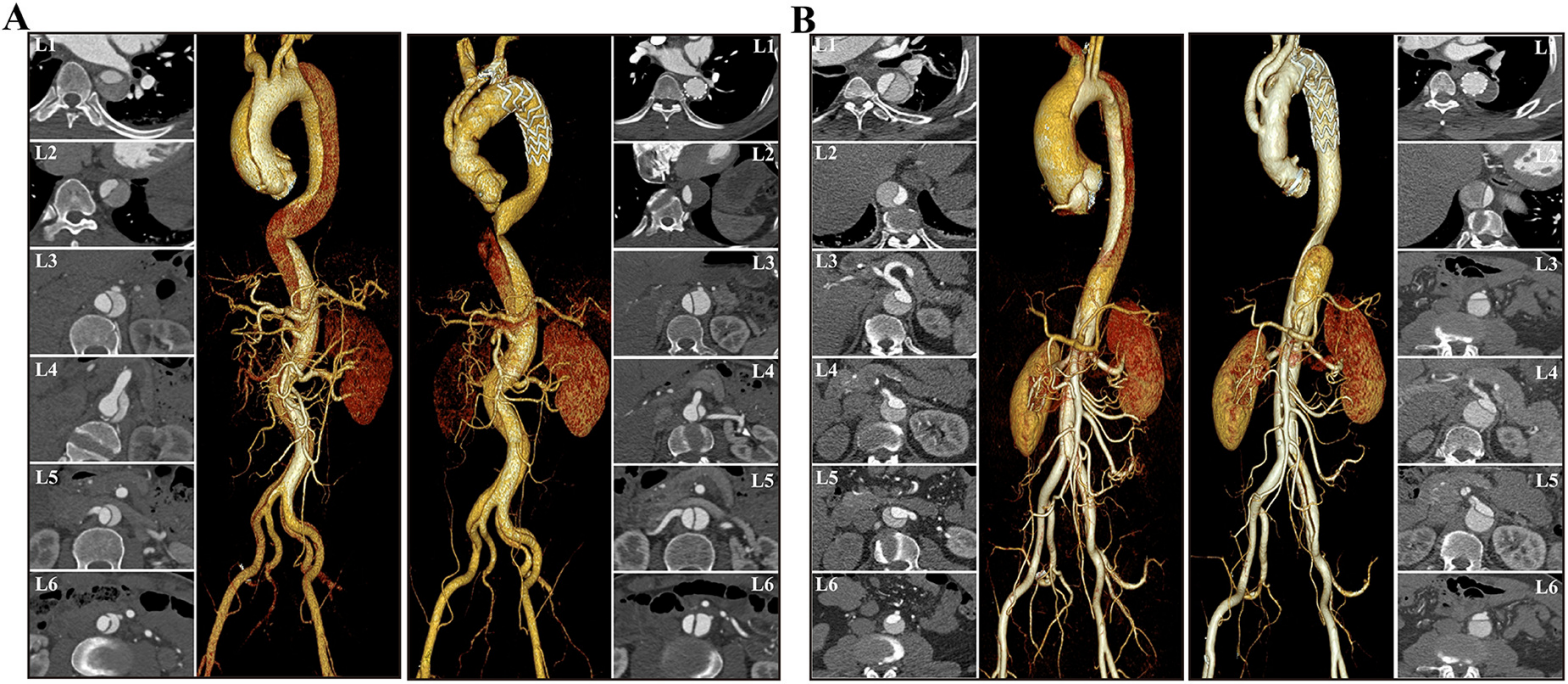
Examples of preoperative and postoperative morphological features of DeBakey I type aortic dissection. The cross-sectional and VR images showed that FL thrombosis was observed at the level of the diaphragm in the non-valve replacement group **(A)**, whereas at the level of the distal stent in the valve replacement group **(B)**. Both groups demonstrate a trend of decreased AL diameter in the proximal descending aorta and increased AL diameter in the distal aorta. VR, volume rendering; AL, aortic lumen; TL, true lumen.

The occurrence of FL thrombosis in various segments of the aorta is detailed in Table 6 and Figure 4. At the last follow-up, the incidence rates of FL thrombosis were 95.8%, 80.6%, 45.8%, 30.6%, 34.7%, and 38.9% for levels L1, L2, L3, L4, L5, and L6, respectively. In general, FL thrombosis rates tended to increase in all segments after surgery in both groups, but statistically significant differences were observed at L1 and L2 levels in the valve replacement group and at L1–L3 levels in the non-valve replacement group. Comparing the postoperative thrombosis rate of the two groups, it was found that only the statistical significance was observed in level L3, and there was no statistical difference between the two groups in other levels. Classification based on the degree of FL thrombosis reveals that as sections closer to the stent, the complete occlusion rate of the FL is higher (67.0% in the valve replacement group and 81.0% in the non-valve replacement group), with statistically significant differences. Figure 5 shows two typical examples of preoperative and postoperative morphological features of DeBakey I type aortic dissection.

**Table 6:** False lumen thrombosis status.

Level of aorta	Whole cohort			Valve Replacement group			Non-valve Replacement group		
	PO (%)	FU (%)	*p* value	PO (%)	FU (%)	*p* value	PO (%)	FU (%)	*p* value
Level 1	7 (9.7%)	69 (95.8%)	<0.000*	1 (3.3%)	29 (96.7%)	<0.000*	6 (14.3%)	40 (95.2%)	0.000*
Level 2	11 (15.3%)	58 (80.6%)	<0.000*	3 (10.0%)	23 (76.7%)	<0.000*	8 (19.0%)	35 (83.3%)	0.000*
Level 3	12 (16.7%)	33 (45.8%)	0.000*	4 (13.3%)	9 (30.0%)	0.209	8 (19.0%)	24 (57.1%)	0.000*
Level 4	14 (19.4%)	22 (30.6%)	0.178	5 (16.7%)	8 (26.7%)	0.532	9 (21.4%)	14 (33.3%)	0.221
Level 5	15 (20.8%)	25 (34.7%)	0.093	7 (23.3%)	10 (33.3%)	0.568	8 (19.0%)	15 (35.7%)	0.087
Level 6	25 (34.7%)	28 (38.9%)	0.730	10 (33.3%)	10 (33.3%)	>0.999	15 (35.7%)	18 (42.9%)	0.503

Note: Data are presented as *n* (%). PO, preoperative. FU, follow up.

*Denotes a significant difference between the samples (*p* < 0.05).

## 4 Discussion

Sun's procedure has been widely adopted as a first-line treatment for DeBakey I type aortic dissection in China, achieving favorable aortic remodeling by covering the entry tear and facilitating subsequent FL thrombosis (20, 21). Aortic remodeling can be influenced by various factors (22), among which the impact of warfarin anticoagulation after aortic valve replacement on medium and long-term outcomes in patients with extensively repaired aortic dissection remains underexplored. This study explores the effects of anticoagulation after aortic valve replacement on medium and long-term vascular remodeling in DeBakey type I aortic dissection. The results showed that post-aortic valve replacement anticoagulation therapy has been proven to affect aortic diameters and FL thrombosis rate in DeBakey type I aortic dissection. However, its impact on aortic remodeling rate is not significant. Overall, anticoagulation therapy appears to be a safe and effective treatment strategy for DeBakey type I aortic dissection. This work is unique in its comprehensive analysis of changes in aortic diameters, aortic remodeling, and FL thrombosis across different segments, emphasizing the importance of continuous aortic monitoring after aortic dissection.

Previous studies have reported that the incidence of thoracic and abdominal aortic expansion 2 years after aortic dissection repair was 21.6% and 33.3%, respectively (23). By 5 years post-surgery, the combined incidence of thoracic and abdominal aortic expansion rose sharply to 62.7% (23, 24). Aortic dilatation is the most common complication of residual aortic dissection following surgical repair. Such complications can lead to persistent FL expansion, the formation of dissecting aneurysms, aortic rupture, or organ ischemia, thereby increasing the risk of reoperation and aorta-related mortality (25, 26). Previous research has identified several strong predictors of aortic expansion after dissection repair, including the number of dissection tears, ongoing FL perfusion, extent of dissection involvement, position of stent coverage, and age over 60 years (11, 27). Some scholars argue that warfarin anticoagulation after aortic valve replacement affects FL thrombosis and remodeling, posing adverse factors for TL formation and FL closure after aortic dissection. In this study, there were no statistically significant differences between the two patient groups in terms of age, number of dissection tears, branches originating from the FL, extent of dissection involvement, aortic tortuosity, or distal position of stent coverage. This research specifically aims to evaluate the impact of anticoagulation after aortic valve replacement on aortic expansion, while mitigating potential confounding factors associated with aortic dissection drivers mentioned above.

Assessment of morphological changes post-aortic surgery, including alterations in AL, TL, and FL diameters, remains a topic of ongoing debate. Consensus has yet to be reached on which specific parameters (such as diameter, area, volume, or the absolute ratio of AL to TL growth) or thresholds (e.g., a 10% or 20% increase) should be used to define aortic remodeling (3, 28). Patterson et al. have demonstrated that diameter measurements correlate well with cross-sectional area and are appropriate tools for assessing aortic remodeling (29). Liu et al. have proposed using a 10% change in diameter as a threshold to define significant alterations in AL measurements (3). Based on this, we adopted this approach and applied this cutoff to diameter measurements (17). Our methodology and cohort are distinctive in several aspects: we conducted multiple measurements at different levels of the aorta rather than at single points, ensured mid- to long- term follow-up (at least 6 months), and collected relatively comprehensive clinical data. Results from this retrospective study indicate varied changes in aortic diameter at different levels. Comparing the preoperative and postoperative diameters of the AL and TL in the two groups, there was an overall increasing trend in both the AL and TL diameter except at the distal level of the stent (i.e., Level L1). Additionally, the degree of change in the proximal level of the AL and TL diameter was greater than that in the distal level. This growth in aortic diameter across multiple areas highlights the ongoing necessity for rigorous, lifelong monitoring of the aorta.

Unlike previous morphological studies that focused solely on changes in AL, this study also considers the outcome of the TL and quantifies the TL diameter at all levels at the follow-up time point. Alongside AL growth, the TL diameter also increases correspondingly, particularly at the distal end of the stent, with statistically significant differences observed. At this location, the TL is replaced by the stent, thereby promoting an increase in the TL. In non-stented areas, TL diameters increase across all levels, although the extent of dilation gradually decreases. These findings indicate that the effectiveness of the stent diminishes gradually as the aortic descends. The changes in the TL help reduce wall shear stress and pressure, thereby improving hemodynamics and preventing further dissection or re-tearing of the intimal flap postoperatively (24). Consequently, for patients with preoperative aortic dissection, particularly those with a spiral tear or collapse of the TL, it is advisable to use adjunctive techniques to extend the stent's range to appropriate positions, thereby improving long-term outcomes while balancing the risk of spinal cord ischemia (26, 30, 31).

It is important to noted that the process of aortic expansion or remodeling following aortic dissection surgery has not yet been fully explained. Evidence suggests that aortic remodeling can impact the fate of the distal aorta, thereby influencing the need for distal reintervention (14, 32). While there is no universally accepted definition or standardized method for measuring aortic remodeling across the literature, it has typically been assessed using cross-sectional and volume index evaluations. Previous studies have reliably shown morphological changes in the thoracic aorta following stent placement (15, 33, 34). This retrospective study showed that positive remodeling was more common in the stented segment, while negative remodeling becomes more probable as one approaches the distal aorta, consistent with prior research findings (5). In contrast, this study demonstrated that preoperative aortic valve replacement did not significantly influence aortic remodeling postoperatively, nor does it increase surgery-related complications. These findings suggest that previous risk assessments regarding the prognostic impact of anticoagulation on aortic dissection may have been overestimated. Overall, anticoagulation therapy following aortic valve replacement appears to yield favorable remodeling outcomes for DeBakey Type I aortic dissection.

Postoperative thrombosis in DeBakey type I aortic dissection is a significant predictor of aortic dilation and long-term survival (31, 35). Under the activation of FL thrombosis formation, downstream aortic remodeling may lead to FL contraction and occlusion (13, 36). After aortic valve replacement, anticoagulation therapy influences FL thrombosis, which can result in the continuous expansion of the distal FL in aortic dissection. This provides the basis for the observation of aortic remodeling based on FL thrombosis formation (37, 38). In the valve replacement group, the incidence of FL thrombosis formation at the distal end of the stent and at the diaphragmatic level was significantly increased postoperatively compared to preoperatively, whereas in the non-valve replacement group, the ratio of thrombosis formation in the FL at the distal end of the stent, diaphragmatic level, and celiac trunk artery level was significantly increased postoperatively, with statistical significance observed in the differences. In the stent segment, TL expands due to the radial force of the stent, simultaneously causing FL contraction or even disappearance, slowing down blood flow into FL, reducing internal pressure, and promoting thrombosis formation in FL (22, 36). In all, the incidence of FL thrombosis formation increased in all patients after surgery compared to preoperative trends. A multicenter registry study in Europe reported thrombosis formation rates of 99.3% and 52.6% in covered and uncovered areas of stent implantation after aortic dissection, respectively, with lower thrombosis formation rates in the abdominal aorta at 13.9% (17, 39). Compared to our study, the thrombosis formation rate in the distal covered area was as high as 96.8%, consistent with previous research results; however, in our study, the thrombosis formation rate in the diaphragmatic level of the thoracic aorta was 79%, and in the abdominal aorta segment it was 30.6%-46.8%, higher than previous research results, which can be explained by the assessment of mid- to long- term FL thrombosis formation status in our study. In general, except for the higher postoperative FL thrombosis formation rate at the distal stent level (100%) in the valve replacement group compared to the non-valve replacement group, the thrombosis formation rates at other levels were significantly higher in the non-valve replacement group than in the valve replacement group. Furthermore, the diversity of thrombosis formation should be considered, as there were distinct differences in the postoperative FL thrombosis status between the two groups (16, 31). The valve replacement group had a significantly higher proportion of absence of thrombosis in the celiac trunk artery segment (*p* = 0.0286) than the non-valve replacement group, with no significant difference in thrombosis status between the two groups at other levels. The findings indicate that anticoagulation after aortic valve replacement indeed affects thrombosis formation in the FL postoperatively. However, it is important to note that thrombosis formation is a dynamic process, and the impact on FL thrombus status may be masked. There is a possibility that partial thrombosis could progressively evolve into malabsorption, with a slight but continuous increase in thrombosis formation. This phenomenon warrants further investigation in future studies.

## 5 Limitations

Our current study has several limitations. The most notable of these include its retrospective nature, small number of participants, and single-center design, all of which may pose potential sources of bias. Firstly, this is a retrospective study, selection bias may influence the results. Secondly, the relatively small sample size, coupled with the fact that all patients were from a single center, limits the statistical power and generalizability of the findings. Thirdly, due to the long-term follow-up period being insufficient, longer follow-up is needed in the future to further accumulate data for evaluating aortic remodeling and surgical outcomes. Fourthly, the term “aortic remodeling” lacks a standardized definition and is widely used in the literature (17), potentially leading to differences in direct comparisons with other studies. In the future, we may attempt to conduct prospective, multicenter, and longer-term follow-up studies to evaluate predictive factors of remodeling status after aortic dissection surgery based on patients' clinical and multimodal imaging data. However, considering our study's results through comprehensive analysis of diameter measurements at multiple levels of the distal aorta and FL status, this research offers high utility in better elucidating the effect of anticoagulation on the aorta remodeling after aortic valve replacement.

## 6 Conclusions

The results of this study showed that there was no significant correlation between the involvement of the aortic valve and the extent of tear at the distal end of DeBakey type I aortic dissection. Surgery involving Sun's procedure combined with aortic valve replacement compared to Sun's procedure alone resulted in longer operative times, increased red blood cell transfusions, partly affected the diameter of the aorta, and the status of thrombus formation in the FL. However, there was no significant impact on the rate of residual aortic remodeling postoperatively. Anticoagulation therapy appears to be a safe and effective treatment strategy for DeBakey type I aortic dissection. For patients who undergo anticoagulation therapy after aortic dissection, regular follow-up imaging is necessary, with particular attention to aortic dilation or thrombosis.

## Data Availability

The original contributions presented in the study are included in the article/Supplementary Material, further inquiries can be directed to the corresponding authors.
